# O043: Early adaptors of a hand hygiene control system

**DOI:** 10.1186/2047-2994-2-S1-O43

**Published:** 2013-06-20

**Authors:** A Lehotsky, M Nagy, P Rona, L Szilagyi, G Weber, T Haidegger

**Affiliations:** 1 Department of Surgical Research and Techniques, Semmelweis University, Hungary; 2 Clariton Ltd, Budapest, Hungary; 3 Dept. of Control Engineering and IT, Budapest University of Technology and Economics, Budapest, Hungary; 4 Austrian Center for Medical Innovation and Tehnology, Wiener Neustadt, Austria

## Introduction

Ignaz Semmelweis discovered the importance of hand hygiene in 1858, and 150 years later, it was the Semmelweis University (SU) in Budapest that first adapted a digital tool to effectively teach proper hand disinfection technique. The Department of Surgical Research and Techniques at SU began to employ a UV dye-based, computer-imaging empowered device after it received the 1st ICPIC Innovation Academy Award.

## Objectives

To introduce objective hand hygiene control in the early stage of medical education.

## Methods

The training of 3rd year MD students at SU starts with the theoretical and practical education of hand disinfection; their performance is assessed early in and at the end of the semester. In the end, all students must proof a perfect hand rubbing to qualify for exams. First in 2011, 377 students were tested and next year, 281 students took part. The hand rubbings were imaged, recorded, and subsequently analyzed to identify error patterns in coverage. Moreover, comparable results from a week-long trial in 2011 from the National University Hospital Singapore (NUH) were also acquired, involving 95 students.

## Results

67% of students completed the rubbing perfectly at first in 2011, while 64.5% passed in the following year. Most errors occurred on the back of the hand, at the tips and the thumb, these responsible for 55% and 52% of errors in 2011 and 2012, respectively. Singapore students presented a 60 % pass rate. Failed students were redirected for education, to acquire a better hand hygiene practice.

## Conclusion

Through objective testing, a clear quality feedback was provided to students. This helped them to correct their errors and should lead to improved practice regarding safe patient care. Participants reported highly positively about the use of the digital assessment system. Furthermore, the device has been employed at major public outreach event with great success, attracting several hundred visitors at the hand hygiene booth.

## Disclosure of interest

None declared.
